# Autoextraction of Permanent Incisors and Self-Inflicted Orodental Trauma in a Severely Burned Child

**DOI:** 10.1155/2015/425251

**Published:** 2015-12-30

**Authors:** Sultan Keles, Gülçin Dogusal, Işıl Sönmez

**Affiliations:** Department of Paediatric Dentistry, Faculty of Dentistry, Adnan Menderes University, 09100 Aydın, Turkey

## Abstract

Autoextraction is one type of self-injurious behaviour. In the literature, self-injurious behaviours are observed in syndromes and genetic conditions. However, to the best of our knowledge, SIB and autoextraction in a severely burned patient have not been reported to date. This report describes the self-inflicted trauma and autoextraction in a severely burned child, and the management of the child during and after burn treatment.

## 1. Introduction

Self-injurious behaviour (SIB) is identified as an activity in which an individual inflicts injury or harm to himself or herself. There are different forms of SIB, including head banging on hard surfaces, such as walls or floors, pinching, scratching, biting of the skin or gums, pulling hair, slapping or punching the face or head, eye poking and cornea scratching, and autoextraction [1].

SIB has been associated with biochemical disorders, syndromes, and genetic conditions, such as Lesch-Nyhan syndrome, De Lange and Tourette's syndromes, learning difficulties, autism, psychological disturbances, and schizophrenia [2, 3]. SIB may also be observed in certain infectious diseases, such as encephalitis, and patients with hereditary sensory and autonomic neuropathy (HSAN), which is characterized by a high tolerance or insensitivity to pain [4, 5].

In the literature, there are various types of self-inflicted behaviours. SIB has been observed in a child affected with Hallervorden-Spatz disease, a rare neurodegenerative condition that is characterized by progressive dystonia, rigidity, and mental retardation [6].

Brahim et al. reported oral and maxillofacial complications, such as lip, hand, tongue biting, and self-extraction, which has been associated with congenital sensory neuropathy and anhydrosis in two cases [7].

Williams reported a 12-year-old child with autistic spectrum disorder, in which twelve of his permanent teeth have been extracted. A six-year-old child with autistic spectrum disorder extracted his deciduous canine tooth [1].

However, to the best of our knowledge, autoextraction of primary and permanent teeth during severe burn treatment has not been reported to date. The aim of this report was to describe a case of a self-inflicted orodental injury and autoextraction of primary and permanent teeth in a 7-year-old child, which may be related to chronic pain caused by a severely burned body.

## 2. Case Report

A 7-year-old boy was admitted to the Intensive Care Department of Adnan Menderes University in Aydin, Turkey, with a diagnosis of third-degree severe burns (67% of his body) due to playing with a gas lighter. The patient had no systemic disease. In the present case, fluid improvement was achieved, and the patient was given systemic antibiotics to prevent high infection risk. A grafting operation was performed on the patient's back to burned areas in his leg [8]. The patient was treated in a hospital setting for 5 months. After he was discharged from the hospital, he had walking disability resulting from his burns.

**Figure 1:**
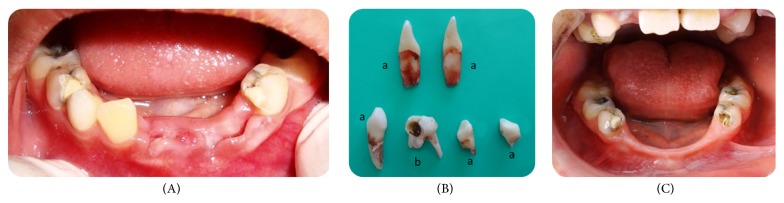
(A) Oral status of the patient on the first visit in the hospital. (B) a: teeth extracted by the patient, b: tooth extracted by the pediatric dentist. (C) Clinical examination (after 5 months).

By the second month of hospitalization, the paediatrician had asked for a consultation from the Faculty of Dentistry because the patient demonstrated teeth grinding and had extracted his own teeth. A paediatric dentist (ISS) visited the patient in the hospital's burn unit. The father of the patient showed the paediatric dentist the autoextracted teeth (Figure 1(B); a). His father reported that he had extracted one or two more teeth prior to this dental examination. In the clinical examination, there were deep caries in teeth 54 and 64. The patient reported severe pain in tooth 54; thus, the teeth were extracted in the hospital under local anaesthesia (Prilocaine, Citanest, AstraZeneca-Eczacıbaşı, Istanbul, Turkey). Two weeks following the extraction, upper and lower impressions were made. A full-coverage lower soft polyvinyl bite guard was constructed on a model of dental stone and fitted postoperatively. Preventive dental advice was given to the father on oral hygiene and care of the appliance, which was to be worn during teeth grinding. Despite the father's specific attention, the patient could not use the bite guard due to chronic pain from the burn treatment.

**Figure 2:**
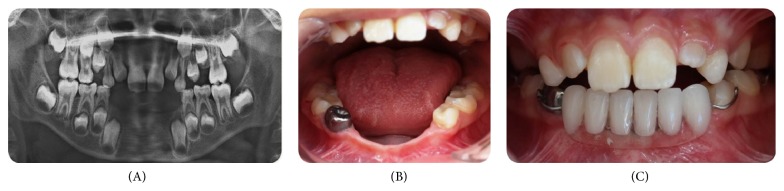
(A) Radiological examination (after 5 months). (B, C) Completed restorations and removable denture.

When the patient was discharged from the hospital after five months, clinical and radiographical examination of the patient was performed in the Faculty of Dentistry, and it was reported that he also extracted teeth 42 and 83 after the last dental examination in the hospital (Figure 1(C)). There were also dental caries in the maxillary and mandibular primary molars. The mandibular right primary first molar was pulpatomized and restored with a stainless steel crown and the remaining primary molars were replaced with compomer restorations. After completion of all of the necessary treatments of the teeth, a partial removable denture was prepared for aesthetic and functional requirements (Figure 2). Follow-up evaluations of the patient continue periodically.

## 3. Discussion

Injuries such as burns cause extensive skin damage, resulting in fluid, protein, and blood loss. Prevention of infection and fluid replacement are integral to the success of the patient and his or her surgical reconstructive procedures. Pain is one of the most important factors that disrupts patient comfort. It is well-known that burned areas and graft operations are highly painful conditions. The consequences of pain may be even more detrimental: disruptions in the development of personality, long-lasting psychological problems, and posttraumatic stress disorders are particularly problematic in burned undermedicated children compared to adults.

SIB was generally connected to diseases with neuropsychiatric symptoms and infectious diseases or the patient was very young in the literature [1–3, 5, 6]. In this case, a systemically healthy and severely burned child who was undermedicated was self-mutilated by extracting his teeth. The authors propose that autoextraction of the teeth may be related to the complicated, painful, and long-lasting burn treatment.

Several investigators have proposed self-injurious behaviour to be a reaction to chronic pain in animals [9]. Several case reports have described self-injurious behaviour derived from neuropathic pain in human adults and children [10]. However, there is no standard method to prevent or treat orofacial self-inflicted injuries. An appropriate treatment plan has been established according to the specific circumstances of each individual case.

Although local, systemic, and psychological factors have been attributed to teeth grinding, its precise aetiology is unknown [11]. In the present case, the self-injurious behaviour resulted in injury to the primary and permanent teeth during teeth grinding, clenching, and autoextraction due to severe pain. Intraoral appliances were used to treat self-inflicted orodental injuries [4]. These appliances may be effective only if the patient tolerates and properly retains the appliance [12–14]. In the present case, we aimed to protect the patient's teeth and oral soft tissue from further damage using a bite guard. However, the patient did not tolerate the appliance.

Next, we completed all restorations of the teeth to eliminate oral causes of the pain in order to reduce severe pain in the patient. Finally, we created a partial removable denture for aesthetic and functional requirements. A partial removable denture is also important for space-maintaining reasons for the following restorations into adulthood.

## 4. Conclusions

Severe pain during burn treatments may result in tooth grinding and self-inflicted orodental trauma. A dentist should supervise such patients during their burn treatment.
